# Adding In Silico Assessment of Potential Splice Aberration to the Integrated Evaluation of *BRCA* Gene Unclassified Variants

**DOI:** 10.1002/humu.22973

**Published:** 2016-04-15

**Authors:** Maxime P. Vallée, Tonya L. Di Sera, David A. Nix, Andrew M. Paquette, Michael T. Parsons, Russel Bell, Andrea Hoffman, Frans B. L. Hogervorst, David E. Goldgar, Amanda B. Spurdle, Sean V. Tavtigian

**Affiliations:** ^1^ Department of Molecular MedicineCHUQ Research CenterQuebec CityCanada; ^2^ Department of Human GeneticsUniversity of Utah School of MedicineSalt Lake CityUtah; ^3^ ARUP LaboratoriesUniversity of Utah School of MedicineSalt Lake CityUtah; ^4^ Department of Oncological Sciences, Huntsman Cancer InstituteUniversity of Utah School of MedicineSalt Lake CityUtah; ^5^ QIMR Berghofer Medical Research InstituteBrisbaneAustralia; ^6^ Netherlands Cancer InstituteAmsterdamThe Netherlands; ^7^ Department of Dermatology, Huntsman Cancer InstituteUniversity of Utah School of MedicineSalt Lake CityUtah

**Keywords:** BRCA1, BRCA2, cancer predisposition, unclassified variant, variant of uncertain significance, rare variant

## Abstract

Clinical mutation screening of the cancer susceptibility genes BRCA1 and BRCA2 generates many unclassified variants (UVs). Most of these UVs are either rare missense substitutions or nucleotide substitutions near the splice junctions of the protein coding exons. Previously, we developed a quantitative method for evaluation of BRCA gene UVs—the “integrated evaluation”—that combines a sequence analysis‐based prior probability of pathogenicity with patient and/or tumor observational data to arrive at a posterior probability of pathogenicity. One limitation of the sequence analysis‐based prior has been that it evaluates UVs from the perspective of missense substitution severity but not probability to disrupt normal mRNA splicing. Here, we calibrated output from the splice‐site fitness program MaxEntScan to generate spliceogenicity‐based prior probabilities of pathogenicity for BRCA gene variants; these range from 0.97 for variants with high probability to damage a donor or acceptor to 0.02 for exonic variants that do not impact a splice junction and are unlikely to create a de novo donor. We created a database http://priors.hci.utah.edu/PRIORS/ that provides the combined missense substitution severity and spliceogenicity‐based probability of pathogenicity for BRCA gene single‐nucleotide substitutions. We also updated the BRCA gene Ex‐UV LOVD, available at http://hci‐exlovd.hci.utah.edu, with 77 re‐evaluable variants.

## Introduction

Clinical resequencing of a high‐risk cancer susceptibility gene such as *BRCA1* (MIM #113705) or *BRCA2* (MIM #600185) may reveal that a patient carries a clearly pathogenic sequence variant. Most pathogenic variants in these genes are either nonsense variants, small insertion, or deletion variants (indels) that create a frameshift, larger gene rearrangements, variants that create a severe splicing aberration, or severely dysfunctional missense substitutions. However, testing may also reveal that a patient carries an UV (VUS for variant of uncertain clinical significance). In *BRCA1* or *BRCA2*, these are usually missense substitutions, in‐frame indels, or sequence variants that fall in the splice junction consensus regions but outside of the canonical GT‐AG dinucleotides. Even silent substitutions could be pathogenic if they have a severe impact on the regulation of mRNA splicing.

A Bayesian “integrated evaluation” or “multifactorial method” has proven to be a relatively successful approach to classification of *BRCA* gene UVs [Goldgar et al., 2004; Easton et al., 2007; Goldgar et al., 2008; Spurdle, 2010; Lindor et al., 2012], and there is now a database dedicated to *BRCA* gene variants that have been classified by this method [Vallée et al., 2012]. This approach is also being applied to variants in other high‐risk cancer genes, notably the mismatch repair genes [Thompson et al., 2013a]. In this approach, UVs are assessed through a Bayesian inference that starts with a prior probability in favor of pathogenicity based on position in the gene and sequence analysis [Easton et al., 2007; Tavtigian et al., 2008]. The prior is updated with observational data from segregation analysis, summary personal and family history analysis, co‐occurrence between UVs and clearly pathogenic sequence variants, and tumor immunohistochemistry and grade. Each type of observational data is expressed as odds or likelihood ratios in favor of pathogenicity. The resulting posterior probability is then converted to one of five qualitative classes, based on defined cut‐points considered to be clinically relevant [Plon et al., 2008].

One limitation to the prior probability is that its sequence analysis component has only been calibrated for missense substitutions and sequence variants that alter the canonical GT‐AG splice junction dinucleotides [Easton et al., 2007; Tavtigian et al., 2008]. Yet, it is well known that sequence variants in the broader proximal splice junction regions can damage function of the wild‐type splice junctions, sequence variants in either the exons or the introns can create de novo splice junctions, and sequence variants in either the exons or introns can alter splice enhancers or splice silencers; all of these classes of sequence variants have the potential to cause aberrant splicing.

The work presented here focuses on calibrating MES [Yeo and Burge, 2004] based analysis of variants in the proximal splice junction region that may damage the function of wild‐type splice junctions, and of exonic variants that may create de novo splice junctions. The analysis covered all exonic bases, plus 20 intronic bases upstream and six intronic bases downstream of each exon, and converts the MES score into a probability of pathogenicity so that it can be included in the integrated evaluation of *BRCA* gene variants.

## Methods

### Dataset

The dataset comprised results of full sequence tests carried out at Myriad Genetic Laboratories, as used previously in Easton et al. (2007) and Tavtigian et al. (2008) for modelling of risk associated with *BRCA1/2* sequence variation. The analyses described here are based on results of full sequence tests of both genes from 68,000 BRACAnalysis subjects of whom 4,867 were reported to carry a pathogenic *BRCA1* variant and 3,561 were reported to carry a pathogenic *BRCA2* variant. For a test to have been performed, a test request form must have been completed by the ordering health care provider, and the form must have been signed by an appropriate individual, indicating that "informed consent has been signed and is on file." The mutation screening data are arranged by sequence variant rather than by subject. The dataset includes nucleotide and amino acid nomenclature specifications for all of the exonic single‐nucleotide substitutions—silent, missense, or nonsense—observed from the 68,000 patient mutations screening set; these are all of the observational data required to calculate the enrichment ratio for single‐nucleotide substitutions (ERS) [Tavtigian et al., 2008].

Analyses of the personal and family history of tested probands to recalculate family history likelihood ratios (FamHx‐LRs) derive from a virtually identical series of subjects used previously [Easton et al., 2007]. However, this dataset also includes frameshifts, in‐frame indels, and sequence variants falling in the intronic portions of the splice junction consensus regions from −20 to +6 of the protein coding exons. We refer to these two overlapping data sets as the B1&2 68K set.

Sequence variant data from the DataBase of Aberrant 3′ and 5′ splice sites (DBASS3 and DBASS5) [Buratti et al., 2011] and the Breast Cancer Information Core (BIC) (https://research.nhgri.nih.gov/projects/bic/index.shtml) were obtained in December 2011; the DBASS data were updated in May 2015.

### Scoring Sequence Variants with MES

MES is a program, based on a maximum entropy model, for scoring the fitness of potential splice donor or splice acceptor sequences [Yeo and Burge, 2004]. Rather than scanning an entire sequence to find the best candidates, the program scores fixed k‐mers (9‐mers with the candidate splice site between the 3rd and 4th nucleotide position to evaluate donor splice sites, 23‐mers with the candidate splice site between the 20th and 21st nucleotide position for acceptors) and then outputs its maximum entropy‐based score for each k‐mer. MES was used to obtain scores for the *BRCA1* and *BRCA2* gene reference sequences. The reference sequences used were: *BRCA1* cDNA NM_007294.3, *BRCA1* genomic NG_005905.2, *BRCA2* cDNA NM_000059.3, and *BRCA2* genomic NG_012772.3. We also used MES to score every possible single‐nucleotide substitution to the coding sequences and proximal splice junction regions plus other individual sequence variants such as in‐frame indels present in the B1&2 68K set. For sequence variants that might damage a wild‐type splice site, this was done by scoring the wild‐type site and the mutated site, with the scoring window set such that the position of the splice junction fell at its normal location.

**Figure 1:**
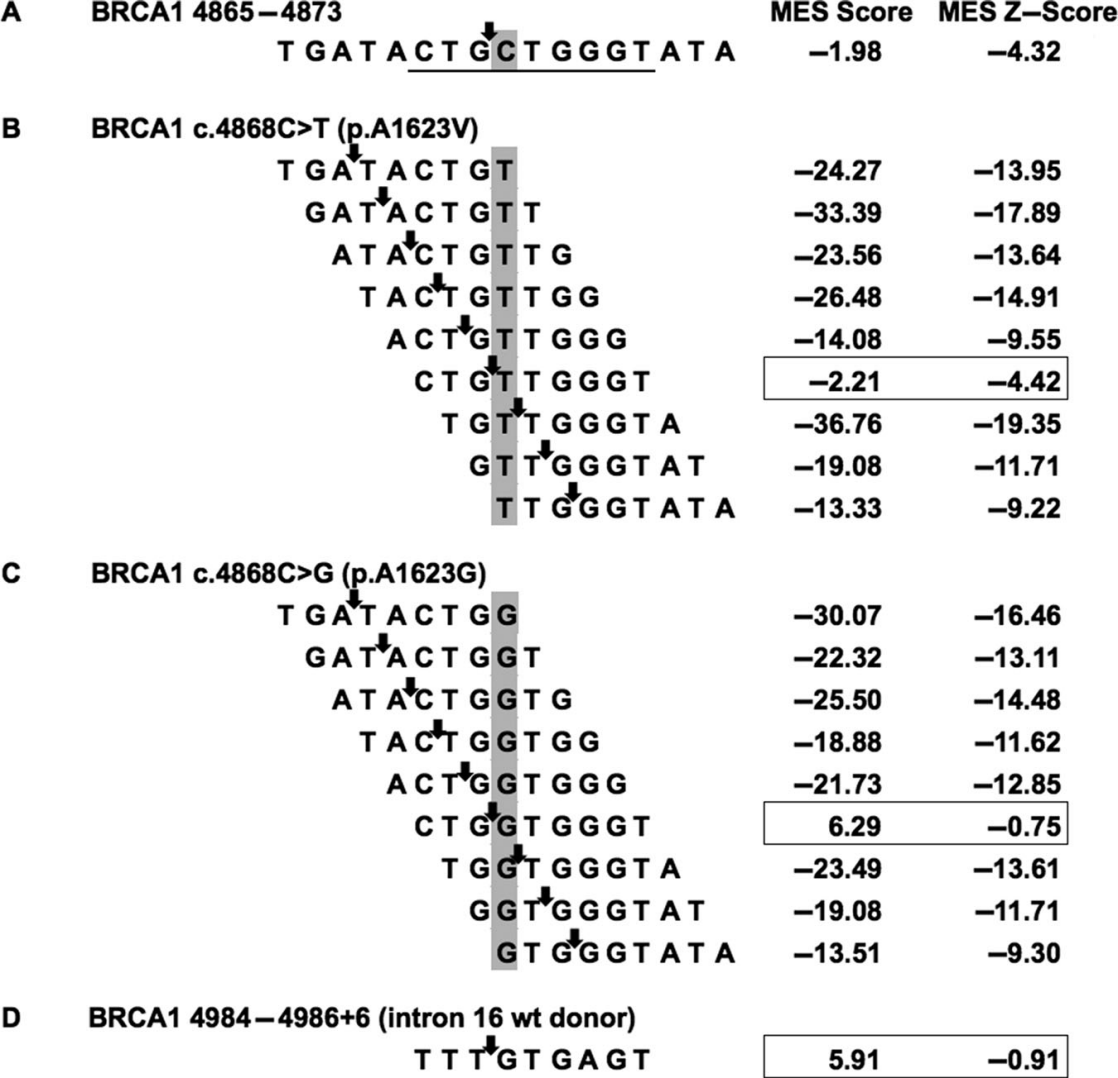
Illustration of the MaxEntScan sliding window approach. **A**: *BRCA1* cDNA reference sequence from 4,865 to 4,873, highlighting nucleotide C4868 in exon 16. **B**: Sliding a MaxEntScan donor window across an innocuous possible substitution, c.4868C>T. **C**: Sliding a MaxEntScan donor window across the de novo donor creating substitution c.4868C>G; note the relatively high MaxEntScan donor score at the sixth frame of the sliding window. **D**: MaxEntScan evaluation of the intron 16 splice donor; note that this score is actually lower than the score of c.4868C>G.

To detect sequence variants that might create a de novo splice junction, including all possible single‐nucleotide substitutions to the *BRCA* gene coding sequences from 20 bp upstream of each coding exon to 6 bp downstream of each coding exon, the following approach was taken. A MES scoring window (i.e., 9 bp for scoring a donor, 23 bp for scoring an acceptor) was slid across the substitution‐bearing sequence such that k k‐mers were scored, with the sequence variant moving from position 1 to position k of the window. Then, the highest score (most fit as a potential splice junction) from each set of k‐mers was recorded in a MySQL database (Figure 1). A java implementation of the MES algorithm was developed and incorporated into an application for scoring variants in a vcf file for damage to known splice sites and introduction of novel splice sites. This is released as part of the open source USeq package at http://useq.sourceforge.net/cmdLnMenus.html#VCFSpliceAnnotator.

### ERS Calculations

The ERS is similar in spirit to the standard population genetics measure *d*_N_/*d*_S_ (*d*_N_ is the nonsynonymous substitution rate and *d*_S_ is the synonymous substitution rate per site), where a positive ratio *d*_N_/*d*_S_ is indicative of positive selection [Yang, 1998]. For each nucleotide in a canonical DNA sequence, there are three possible single‐nucleotide substitutions. However, these substitutions are not equally likely to occur because of differences in the underlying substitution rate constants. Using the dinucleotide substitution rate constants given by Lunter and Hein (2004), averaging sense and antisense orientations, we can estimate a relative substitution rate for every possible single‐nucleotide substitution to a DNA sequence, *r*_i_. The probability that a new sequence variant (i.e., a new germline sequence variant at the moment that it comes into existence) will fall into a particular algorithmically defined class *c* is given by the ratio of the sum of the relative substitution rates of the variants belonging to the class *c* divided by the sum of all relative substitution rates (For this discussion, an “algorithmically defined class” of variants is a class of variants that can be unambiguously specified by an algorithm. One example could be, given a specified protein multiple sequence alignment, all substitutions that fall at an invariant position in the alignment and have a Grantham score ≥65. Another could be all substitutions that fall at the last nucleotide of a protein coding exon.):

(1) pc=∑iεcri∑ all _iri

Hence, under the null hypothesis of no selection, we can obtain from the total number of variants observed in a mutation screening study, *o*_T_, the number expected in any class, $e_{C}=p_{C}\times o_{T}$, and compare this to the actual number observed, *o*_c_. Thus, in general, we define the ERS for any class of substitutions c as the observed/expected ratio for that class normalized by the same ratio for silent (i.e., synonymous) substitutions but excluding the few silent substitutions that are likely to be spliceogenic:

(2) $$\mathrm{ERS}(c)=\frac{o_{c}}{e_{c}}/\frac{o_{s}}{e_{s}}=\frac{o_{c}}{p_{c}}/\frac{o_{s}}{p_{s}}$$

### Summary Family History‐Based Risk Estimates

Sequence variants were stratified by MES scores. Then, all of the summary personal/family histories of the subjects who carried the variants within a particular stratum (after data cleaning as mentioned below) were used to estimate α, the proportion of variants within that stratum that were pathogenic, using the heterogeneity likelihood ratio defined in Easton et al. (2007). Approximate 95% confidence intervals for the heterogeneity proportion were obtained by finding the values α_L_ and α_U_ for which the overall likelihood differed from that at α by an amount equivalent to a likelihood ratio test significant at the 0.05 level [Easton et al., 2007].

### Data Cleaning

One potential confounder in our analyses is that a sequence variant can belong to two algorithmically defined groups at once; for example, a single‐nucleotide substitution can cause a missense substitution that is likely to damage protein function, and the same nucleotide substitution can be likely to create a de novo splice donor. In general, for ERS calculations, likely spliceogenic nucleotide substitutions that are either silent or create missense substitutions that are predicted to be neutral from a missense loss of function point of view were placed in the likely spliceogenic class and withdrawn from the likely neutral class. For FamHx‐LR calculations, likely spliceogenic sequence variants that are also likely to be pathogenic because they create likely damaging missense substitutions were withheld from the analyses. This is because their presence in the calculations could confound the FamHx‐LRs in an upwardly biased fashion. On the other hand, likely spliceogenic sequence variants that would probably lead to in‐frame indels in regions of *BRCA1* or *BRCA2* where neither severe missense substitutions nor in‐frame indels are thought to confer high risk of breast or ovarian cancer (e.g., outside of the BRCA1 RING and BRCT domains and outside of the BRCA2 DNA‐binding and PALB2‐binding domains [Easton et al., 2007; Tavtigian et al., 2008]) were withheld from the analyses because their presence in the calculations could confound the FamHx‐LRs in a downwardly biased fashion.

Simple proportions and 95% confidence intervals were estimated in STATA 11.0 using its exact binomial confidence interval calculator.

### Locus‐Specific Database URLs

The Breast Cancer Information Core database:

https://research.nhgri.nih.gov/projects/bic/index.shtml

The *BRCA1* and *BRCA2* prior probabilities database:

http://priors.hci.utah.edu/PRIORS/

The *BRCA1* and *BRCA2* classified variants “Ex‐UV” LOVD:

http://hci‐exlovd.hci.utah.edu

[Please note that this Ex‐UV database supersedes a similar database located at http://brca.iarc.fr/LOVD.]

## Results

To get an approximate idea of MES scores indicative of sequence variants that damage wild‐type splice junctions, and of sequence variants that create de novo splice junctions, we mined the DBASS3 and DBASS5 database [Buratti et al., 2011]. This database holds records of published variants (single‐nucleotide variants and indels) that have an impact on splicing of disease susceptibility genes (e.g., congenital hypothyroidism with *SLC5A5* gene, hypofibrinogenemia with *FGB* gene). Our testing dataset was composed of 201 different genes.

On average, sequence variants that damaged function of a wild‐type splice junction reduced the MES scores of those junctions by more than two standard deviations of the average MES score of a wild‐type splice junction (Table 1). Similarly, sequence variants that created a de novo junction raised the MES score of the underlying wild‐type (nonspliceogenic) k‐mer sequence by more than two standard deviations of the average MES score of a wild‐type splice junction, resulting in MES scores for the de novo junctions that are quite comparable to the MES scores of wild‐type splice junctions in the data set. While it is understandable that the two DBASS data sets contained fewer examples of sequence variants that create de novo splice sites than that damage wild‐type splice sites, we also note that there were fewer examples of de novo acceptors (12) than of de novo donors (65); this holds also for *BRCA1* and *BRCA2* variants.

**Table 1:** MaxEntScan Scores for Spliceogenic Variants from DBASS3 and DBASS5 Compared with Scores for Wild‐Type Splice Junctions for Disease Genes

		MaxEntScan score for wild‐type splice junction^a^	MaxEntScan score for variant sequence damaging splice junction function^b^	MaxEntScan score for wild‐type k‐mer sequence underlying de novo sites^a^	MaxEntScan score for variant k‐mer sequence creating de novo sites^b^
	Number of sequence variants	Ave (SD)	Ave (SD)	Ave (SD)	Ave (SD)
DBASS5 wild‐type donors^a^	336	8.02 (2.10)	0.59 (2.88)	n/a	n/a
DBASS3 wild‐type acceptors^a^	240	8.21 (2.74)	1.53 (3.54)	n/a	n/a
DBASS5 de novo donors	71	7.57 (2.42)	n/a	1.47 (4.94)	7.29 (3.63)
DBASS3 de novo acceptors	12	7.14 (2.26)	n/a	1.90 (3.62)	8.33 (2.39)
*BRCA1*, *BRCA2*, *ATM* wild‐type donors	110	8.02 (2.31)	n/a	n/a	n/a
*BRCA1*, *BRCA2*, *ATM* wild‐type acceptors	110	7.98 (2.44)	n/a	n/a	n/a

a Scores for the relevant wild‐type splice junction k‐mer of exons included in the analysis.

b Scores for the k‐mer containing spliceogenic variants from DBASS. Excludes scores for *BRCA1*, *BRCA2*, and *ATM*.

Asking whether *BRCA* gene splice junctions fall in the same MES score range as the junctions recorded in DBASS, we determined MES scores for the reference sequence wild‐type splice junctions of the canonical coding exons of *BRCA1* and *BRCA2*. To increase the number of wild‐type splice junctions from known breast cancer susceptibility genes, we also included *ATM* (MIM #607585) (Table 1, last two lines). We note that the average MES scores for the *BRCA* splice junctions and the wild‐type splice junctions reported in DBASS are mutually within one‐half standard deviation of each other.

**Figure 2:**
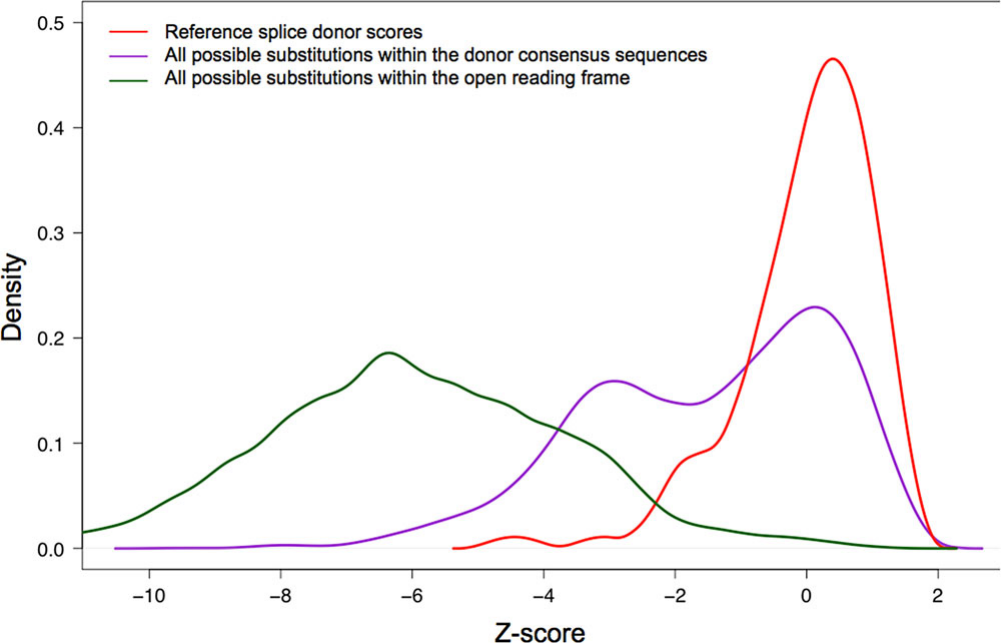
Distribution of MaxEntScan donor splice‐site scores. **Red**: Reference sequence splice donors. **Purple**: All possible single‐nucleotide substitutions to the reference splice donors. **Green**: All possible single‐nucleotide substitutions to the open‐reading frames of *BRCA1* and *BRCA2*.

**Figure 3:**
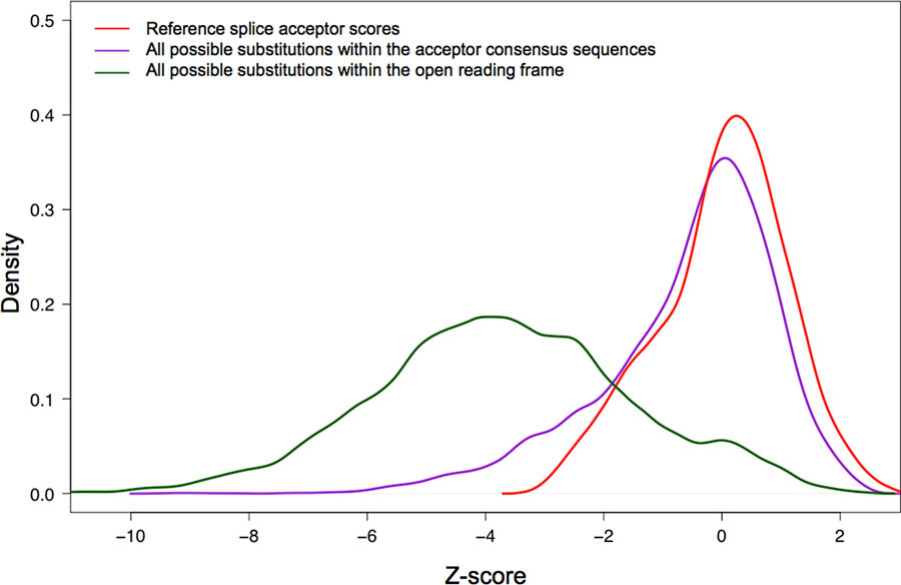
Distribution of MaxEntScan acceptor splice‐site scores. **Red**: Reference sequence splice acceptors. **Purple**: All possible single‐nucleotide substitutions to the reference splice acceptors. **Green**: All possible single‐nucleotide substitutions to the open‐reading frames of *BRCA1* and *BRCA2*.

One difficulty in interpreting MES scores is that they are not standard. The program computes maximum entropy of a sequence; the scoring range is not particularly human interpretable and there are no fixed limits to the highest or lowest possible scores. One of the lowest scores that we observed for a possible substitution in the entire *BRCA1/2* splice‐site prediction dataset was ∼−46, and the highest score that we observed for a wild‐type splice junction was ∼+11. The ranges also appeared to be slightly different for donors and acceptors. To standardize the scores, and noting that the scores for wild‐type splice junctions are approximately normally distributed, we converted the raw MES scores to z‐scores based on the average and standard deviations of MES scores for the canonical protein coding exons of *ATM*, *BRCA1*, and *BRCA2*. This was done separately for donors and acceptors (red curves in Figs. 2 and 3, respectively).

### Calibration of Effects of Sequence Variation in Splice Junction Consensus Sequences

We then examined sequence variation in the splice junction consensus sequences. The distribution of MES scores for all possible single‐nucleotide substitutions to the splice donors is displayed on Figure 2 (purple curve). The curve is bimodal, with about half of the variants causing a notable drop in MES score. In contrast, when we examined the distribution of MES scores for all possible single‐nucleotide substitutions to the splice acceptors, we found that most single‐nucleotide substitutions altering wild‐type acceptor sequences have little effect on the MES score (Fig. 3, purple curve).

In Table 2, we summarize the number of sequence variants that damage wild‐type splice sites (from DBASS, excluding *BRCA1/2* variants) and single‐nucleotide substitutions that damage wild‐type *BRCA1* or *BRCA2* splice sites (from the Breast cancer Information Core [BIC]) as a function of MES z‐score. We also summarize the total number of all possible single‐nucleotide substitutions to the *BRCA1* and *BRCA2* splice consensus regions (9 mer for donor site and 23 mer for acceptor site), and the fraction of these reported in the BIC, as a function of MES z‐score. Clear trends in the data are that the number of reported spliceogenic variants increases in both DBASS and BIC as the MES z‐score decreases, and the fraction of possible *BRCA1/2* variants that are actually reported in BIC follows the same pattern. For variants that damage the splice donors, the increase appears to begin as the MES z‐score drops below 0.0, whereas for acceptors the increase appears to begin as the MES z‐score drops below +0.5.

**Table 2:** MaxEntScan *Z*‐Score‐Based Assessment of Sequence Variants That Damage Wild‐Type Splice Junctions, Recorded in DBASS5, DBASS3, or the BIC

MES *z*‐score interval	DBASS5^a^	DBASS5 (%)	BIC donors^b^	#Possible *BRCA1* or *BRCA2* single‐nucleotide substitutions	Possible *BRCA1* or *BRCA2* single‐nucleotide substitutions reported in BIC (%)
Potential splice donor damage					
(1.5, +inf)	0	0.00	0	6	0.00
(1, 1.5]	0	0.00	0	77	0.00
(0.5, 1]	2	0.74	0	125	0.00
(0, 0.5]	1	0.37	0	161	0.00
(−0.5, 0]	2	0.74	1	133	0.75
(−1, −0.5]	2	0.74	1	99	1.01
(−1.5, −1]	11	4.06	3	104	2.88
(−2, −1.5]	15	5.54	3	58	5.17
(−2.5, −2]	36	13.28	8	126	6.35
(−inf, −2.5]	202	74.54	49	353	13.88
Potential splice acceptor damage					
(1.5, +inf)	1	0.55	0	202	0.00
(1, 1.5]	1	0.55	0	238	0.00
(0.5, 1]	1	0.55	0	438	0.00
(0, 0.5]	5	2.75	0	530	0.00
(−0.5, 0]	5	2.75	0	489	0.00
(−1, −0.5]	7	3.85	0	339	0.00
(−1.5, −1]	8	4.40	3	314	0.96
(−2, −1.5]	27	14.84	6	243	2.47
(−2.5, −2]	36	19.78	9	151	5.96
(−inf, −2.5]	91	50.00	39	368	10.60

a *BRCA1* and *BRCA2* sequence variants recorded in DBASS were removed to avoid double counting.

b Sequence variants deposited in the BIC (http://research.nhgri.nih.gov/bic/) that are located at the *BRCA1* or *BRCA2* native donor and acceptor sites. Evidence for spliceogenicity is not recorded in BIC for all these sequence variants.

Next, we divided contiguous intervals of z‐score range into an ordered series of four qualitative strata. For donor variants, we started with a stratum 1 of *z* ≥ 0 and added strata 2–4: −1 ≤ *z*<0, −2 ≤ *z* ← 1, and *z* ← 2. For acceptor variants, stratum 1 was *z* ≥ 0.5 and strata 2–4 were −0.5 ≤ *z*<0.5, −1.5 ≤ *z* ← 0.5, and *z* ← 1.5, respectively. For both donors and acceptors, we added a stratum 0 for variants that improved (increased) the MES score of the splice junction and removed such variants from the z‐score category into which they would otherwise fall. Using familial history of cancer as a surrogate for genetic risk to measure risk association with potential damage to a wild‐type splice junction as a function of MES score, we then estimated the proportion of variants within the extreme strata that were pathogenic [Easton et al., 2007]. For donor variants, the combination of strata 0 and 1 had a proportion pathogenic of 0.00 (95% CI 0.00–0.36); in clear contrast, stratum 4 had an estimated proportion pathogenic of 1.00 (95% CI 0.78–1.00). For acceptors, the combination of strata 0 and 1 had a proportion pathogenic of 0.00 (95% CI 0.00–0.06); in clear contrast, stratum 4 had a proportion pathogenic of 0.93 (95% CI 0.71–1.00). Two important points emerging from this level of analysis were: (1) the 95% confidence intervals of the stratum 0/1 and stratum 4 groupings were clearly nonoverlapping, providing unambiguous evidence that MES scores of donor and acceptor sequence variants are predictive of pathogenicity, and (2) as the point estimates for the donor and acceptor stratum 0/1 groupings were mutually within each other's confidence intervals, and the point estimates for the stratum 4 donor and acceptor categories were within each other's confidence intervals, it would make sense to combine donor and acceptor z‐score strata into qualitative categories.

To add in strata 2 and 3, we first examined three different two‐stratum partitions: strata 0–3 versus stratum 4; strata 0–2 versus strata 3 and 4; and strata 0 and 1 versus strata 2–4. Of these, the first grouping had a 4.7‐fold better likelihood than the second grouping and a 250‐fold better likelihood than the third grouping. We then examined two partitions of the strata into three groups: strata 0–2 versus stratum 3 versus stratum 4 and strata 0 and 1 versus 2 and 3 versus 4. The latter grouping fit marginally better. We then compared the better 3‐group partition versus the best two‐group partition and found that the three‐group partition fit the data significantly better (*X* ^2^ = 12.67, df =1, *P* = 0.00037). The estimated proportion of pathogenic variants in these three groups are shown in Table 3, as are the calculated splicing priors for pathogenicity per group.

**Table 3:** Qualitative *Z*‐Score Ranges and Probabilities of Pathogenicity for Potential Damage to Reference Splice Junctions

Qualitative category	MES *z*‐score range	Alpha	95% CI	Number of variants used	Prior probability
Improved	Score improved versus reference sequence	0.00	(0.00, 0.08)	46	0.04
Minimal	Donor: *z* > 0	0.00	(0.00, 0.08)	24	0.04
	Acc: *z* > 0.5				
Moderate	Donor: −2 ≤ *z* ≤ 0	0.34	(0.15, 0.55)	66	0.34
	Acc: −1.5 ≤ *z* ≤ 0.5				
High	Donor: *z* < −2	0.97	(0.82, 1.00)	94	0.97
	Acc: *z* < −1.5				

In defining these three qualitative categories, we note that there are a few wild‐type splice junctions in *BRCA1* and *BRCA2* that have very low splice fitness scores. For example, the BRCA1 intron 7 splice acceptor has a MES score of just 3.71, resulting in a z‐score of −1.75; consequently, any sequence variant in this acceptor that lowers its score at all would fall in the high probability of pathogenicity category and receive a prior probability of 0.97. Out of caution, we add the criterion that for variants falling in acceptors with wild‐type scores below *z* = −1.0 or donors with wild‐type scores below *z* = −1.5, variants must reduce the splice junction *z*‐score by at least 0.5 in order to be placed in the high probability of pathogenicity category. The main consequence of this rule will be to prevent T>C or C>T substitutions in acceptors with low‐reference sequence MES scores, which are often innocuous, from receiving high prior probabilities of pathogenicity.

### Calibration of Effects of Exonic Sequence Variation That May Create De Novo Splice Junctions

Sequence variants within an exon can create de novo splice donors or de novo splice acceptors, which can in turn disrupt the structure of the mRNA and thus the protein. To assess the bulk potential for sequence variants that would create de novo splice junctions within the open‐reading frames of *BRCA1* and *BRCA2*, we examined the distribution of the highest possible MES splice donor scores and splice acceptor scores for all possible single‐nucleotides substitutions (excluding substitutions within three bp of the end of an exon) to the open‐reading frames of these genes. These are summarized by the green curves in Figures 2 and 3, respectively. The vast majority of possible substitutions have MES scores that are much lower than those for functional (wild type) splice junctions, but at the high end of the score distribution, there is a tail of scores for possible substitutions that are similar to those of functioning splice junctions.

**Figure 4:**
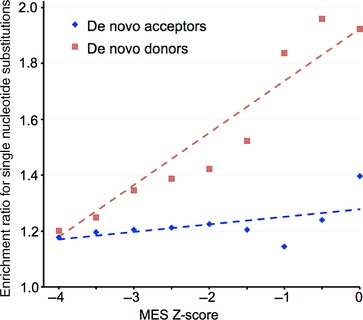
Enrichment for observed exonic substitutions as a function of MaxEntScan de novo donor or de novo acceptor scores. **Red**: Analysis of de novo donor enrichment as a function of MES donor score. **Blue**: analysis of de novo acceptor enrichment as a function of MES acceptor score.

If elevated MES scores for exonic sequence variants are indicative of the creation of de novo splice junctions that are often pathogenic, such variants should be enriched in the Myriad B1&2 68K data set. Excluding variants in the first two or last two nucleotides of the coding exons, 1,698 of the possible 34,840 missense substitutions to *BRCA1* and *BRCA2* are actually observed in the Myriad B1&2 68K data set. Data from these variants were used to estimate the ERS as a function of MES score separately for potential de novo donors and de novo acceptors (Figure 4); this analysis provides a test of the hypothesis that elevated MES *z*‐scores are indicative of aberrant splicing and thus pathogenicity. Because the data set includes missense substitutions that are pathogenic because of missense dysfunction, which should be randomly distributed with respect to the MES *z*‐score, the baseline ERS is inflated to ∼1.2. For the analysis of potential de novo donors, there is a hint of an increased ERS as the MES *z*‐score exceeds −3.0 and greater evidence as the score exceeds −1.0; indeed, a trend test reveals *P* = 2.4×10^−5^ against the hypothesis that the underlying ERS data are drawn from a trendless series. In contrast, the analysis of potential de novo splice acceptors there is at most a hint of a rise in the ERS when the MES *z*‐score reaches or exceeds 0.0, and a trend test does not reveal significant evidence for an increase in the ERS as a function of MES *z*‐score (*P* = 0.07).

Following the same approach described above for variants falling in and around splice donors and acceptors, we then used FamHx‐LR analyses to estimate the proportion of pathogenic variants among exonic substitutions with relatively high MES scores. For these analyses, we used the mirror image of the qualitative categories that emerged from our donor and acceptor damage analyses, that is, the most likely deleterious de novo donor category (increased potential to create a de novo donor) that we defined was *z* ≥ 0, the intermediate category (moderate potential) was −2 ≤ *z*<0, and the least likely (null/weak/low potential) was *z* ← 2. In setting up this analysis, we noted that there were a few sequence variants in the two lower *z*‐score categories with MES scores that are actually higher than the wild‐type splice donor for the exon in which they fell. These were promoted from their qualitative category to the next higher category. For example, a variant in the middle of *BRCA2* exon 16, c.7709A>C, potentially creates a de novo donor with a *z*‐score of −0.63, thus falling in the “moderate” category. The wild‐type donor of exon 16 has a *z*‐score of −1.44. Therefore, this variant is moved to the “increased” category. The resulting summary family history likelihood ratio point estimates for the three categories were 0.01, 0.30, and 0.64, and the result for the *z* ≥ 0 category was independently significant (95% CI 0.06–0.98) (Table 4).

**Table 4:** Qualitative *Z*‐Score Ranges and Probabilities of Pathogenicity for Creation of Exonic De Novo Donors That Would Either Create a Frameshift or Alter a Key Functional Domain

Qualitative category	MES *z*‐score range	Alpha	95% CI	Number of variants used	Prior probability
Weak/null and low^a^	*z* < −2	0.01	(0.00, 0.04)	977	0.02
Moderate^a^	−2 <= *z* < 0	0.30	(0.00, 0.88)	7	0.30
Increased	*z* ≥ 0	0.64	(0.06, 0.98)	8	0.64

a Potentially spliceogenic sequence variants that have a higher MES score than the wild‐type donor for their exon are promoted to the next more severe qualitative category.

As might be predicted from Figures 3 and 4, FamHx‐LR analyses of the groups of exonic variants that had some potential to create de novo splice acceptors did not detect any evidence of increased risk (data not shown).

As was the case in our earlier calibration of Align‐GVGD for evaluating *BRCA1* and *BRCA2* key functional domain missense substitutions [Tavtigian et al., 2008], the proportion of potentially spliceogenic variants estimated to be pathogenic based on the FamHx‐LR analyses can be used as prior probabilities in favor of pathogenicity for integrated evaluations of *BRCA* gene sequence variants. Because priors of 0.00 cannot be used in a Bayesian calculation, and in the interest of making the priors for categories with initial point estimates of 0.00 or 0.01 slightly more conservative, we reassign these to the midpoint of their confidence intervals (Table 3 and 4, rightmost column).

### Correlation with Published Assays of Spliceogenicity for *BRCA1* and *BRCA2* Sequence Variants

To assess how well the splice priors given in Tables 3 and 4 correlate with spliceogenicity assays on *BRCA1* and *BRCA2* sequence variants, we collated data from 73 papers containing interpretable spliceogenicity assays on variants from these genes. The data included one or more assays on 92 acceptor variants, 116 donor variants, and 239 exonic variants that were not predicted to damage either a donor or an acceptor (Table 5; Supp. Table S1). Although some publications provided estimates of transcript ratios based on end‐point PCR, it should be noted that none of these assays were quantitative in the true sense. This is aberration severity as defined below is likely overestimated based on the available reported data; transcript proportions were calculated or estimated from endpoint PCR from at best semiquantitative reactions, and many of the reported aberrations are deletion transcripts that would amplify more efficiently than the longer wild‐type transcripts in a competitive PCR reaction.

**Table 5:** Summary of Published *BRCA* Gene Sequence Variant Spliceogenicity Assay Results

	No aberration	Aberration	Percentage with aberration (95% CI)
Acceptor variants			
High	0	43	100.0 (88.0–100.0)^a^
Moderate	17	13	43.3 (25.5–62.6)
Minimal or improved	18	1	5.3 (0.1–26.0)
Donor variants			
High	2	67	97.1 (89.9–99.6)
Moderate	5	34	87.2 (72.6–95.7)
Minimal or improved	7	1	12.5 (0.3–52.7)
Combined donor and acceptor variants			
High	2	110	98.2 (93.7–99.8)
Moderate	22	47	68.1 (55.8–78.8)
Minimal or improved	25	2^b^	7.4 (0.9–24.3)
De novo donor variants			
Increased	4	9	69.2 (38.6–90.9)
Moderate	2	2	50.0 (6.8–93.2)
Weak/null and low	187	35	15.8 (11.2–21.2)

a Added one discordant observation in order to estimate a 95% confidence interval.

b One of the variants in this category, *BRCA1* c.591C>T, is IARC class 1, neutral [Dosil et al., 2010; de la Hoya et al, submitted].

[Friedman et al., 1994, 1995; Gayther et al., 1995; Petrij‐Bosch et al., 1997; Xu et al., 1997; Hoffman et al., 1998; Mazoyer et al., 1998; Fetzer et al., 1999; Ozcelik et al., 1999; Pyne et al., 1999; Santarosa et al., 1999; Scholl et al., 1999; Hartikainen et al., 2000; Pyne et al., 2000; Laskie Ostrow et al., 2001; Vega et al., 2001; Claes et al., 2002; Fackenthal et al., 2002; Howlett et al., 2002; Krajc et al., 2002; Meindl, 2002; Agata et al., 2003; Claes et al., 2003; Campos et al., 2003; Hofmann et al., 2003; Keaton et al., 2003; Yang et al., 2003; Brose et al., 2004; Sharp et al., 2004; Tesoriero et al., 2005; Bonatti et al., 2006; Chen et al., 2006; Chenevix‐Trench et al., 2006; Beristain et al., 2007; Ang et al., 2007; Anczukow et al., 2008; Bonnet et al., 2008; Farrugia et al., 2008; Goina et al., 2008; Kwong et al., 2008; Machackova et al., 2008; Spearman et al., 2008; Caux‐Moncoutier et al., 2009; Gutierrez‐Enriquez et al., 2009; Li et al., 2009; Vreeswijk et al., 2009; Willems et al., 2009; Dosil et al., 2010; Gaildrat et al., 2010; Hansen et al., 2010; Rouleau et al., 2010; Sanz et al., 2010; Steffensen et al., 2010; Walker et al., 2010; Whiley et al., 2010; Brandao et al., 2011; Thery et al., 2011; Whiley et al., 2011; Zhang et al., 2011; Acedo et al., 2012; Gaildrat et al., 2012; Houdayer et al., 2012; Joose et al. 2012; Menendez et al., 2012; Thomassen et al., 2012; Wappenschmidt et al., 2012; Colombo et al., 2013; Di Giacomo et al., 2013; Parsons et al., 2013; de Garibay et al., 2014; Santos et al., 2014; Whiley et al., 2014a; Acedo et al., 2015; Ahlborn et al., 2015].

The category with the proportionately greatest disparity between the probability of pathogenicity and rate of spliceogenicity was de novo donor weak/null and low. For this category, the probability of pathogenicity was 0.01 (0.00–0.04) versus a spliceogenicity rate of 0.158 (0.11–0.21). Delving more deeply into the published splice assay results, of 35 weak/null and low variants with a reported aberration, 32 were published with an RT‐PCR or minigene assay from which it was possible to estimate the contribution of the mutant allele to a reference transcript (Supp. Table S2). To evaluate these, we used the proportion of frameshifted transcripts produced from the nonpathogenic *BRCA1* allele c.[594‐2A>C;641A>G], ∼70%–80% (de la Hoya et al., submitted), as a guide toward a definition of a severe splicing aberration. To err on the conservative side, we then set >60% aberrant splicing from the variant allele (if the reported result was semiquantitative) or a visibly strong majority of aberrant splicing from the variant allele (if a qualitative result had to be estimated from a gel) as a standard for a severe splicing aberration. Of the 32 evaluable variants, only seven met this splice defect severity criterion (Supp. Table S2). Taken together, a splice aberration rate of 0.158 multiplied by a severe aberration ratio of 0.219 (seven of 32) results in an overall severe splice aberration rate of 0.035 for this category—which is within the 95% confidence interval for the category's probability of pathogenicity.

The other category with a notably higher rate of spliceogenicity than probability of pathogenicity was moderate donor damage. For this category, the probability of pathogenicity was 0.34 (0.15–0.55) versus a splice aberration rate of 0.87 (0.73–0.96). Examining the published data on moderate donor variants with a splice aberration, 22 could be evaluated for severity as defined above (Supp. Table S3). For 17 of these (77%), the aberration was reported to be severe. However, two of the variants with a severe splice aberration (*BRCA1* c.4484G>T and *BRCA2* c.316+5G>C) produce at least one transcript isoform with an in‐frame deletion that does not alter any domain already proven to harbor pathogenic missense substitutions. In addition, another two of these variants produced 17%–20% of canonical transcript in the semiquantitative assay cited (*BRCA1* c.5072C>T and *BRCA2* c.8486A>T) [Houdayer et al., 2012; Santos et al., 2014]. If these latter four variants are actually not pathogenic, then the rate of severe pathogenic splice aberrations in the category would fall to 13/22 (59%), just slightly above the 95% confidence interval of our estimated probability of pathogenicity.

Finally, combining across acceptor and donor variants, we note that there were two splice aberrations reported in the minimal or improved category. One of these, *BRCA1* c.591C>T is considered to be a neutral variant that upregulates naturally occurring in‐frame isoforms [Dosil et al., 2010]. The other *BRCA2* c.68‐7delT leads only to partial skipping of exon 3, again, a transcript which is also seen in controls [Santarosa et al., 1999].

### Expert Knowledge Added to the Prior Probabilities Database

Beyond combining prior probabilities from missense substitution severity and spliceogenicity, we added three expert knowledge elements to the *BRCA1/2* prior probabilities database.

First, we have annotated that substitutions of the translation initiation methionines of *BRCA1* and *BRCA2* have high prior probability of pathogenicity. For BRCA1, this is because the first in‐frame methionine codon (p.M18) falls well within the RING domain, and the resulting N‐truncated protein would delete several residues that are important for the BARD1 interaction [Starita et al., 2015]. For *BRCA2*, this is because there are several out‐of‐frame ATGs located in the mRNA upstream of the first in‐frame methionine codon (p.M124); some of these have a high‐enough translation initiation rate that very little protein synthesis originates from p.M124 [Parsons et al., 2013].

Second, we now know that the prevalence of a *BRCA1* delta exon 9–10 transcript, which encodes a functional protein, is high enough that neither spliceogenic nor protein truncating variants in *BRCA1* exons 9 or 10 have a high probability to be pathogenic [Colombo et al., 2014, Rosenthal et al., 2015, de la Hoya et al, submitted]. To accommodate the observation, we set a ceiling for the probability of pathogenicity at 0.50 for variants in these two exons or their proximal splice junction regions.

Third, we also know that variants that cause skipping of *BRCA2* exon 12, which results in an in‐frame deletion, do not have a high probability to be pathogenic [Li et al., 2009]. As above, we set a ceiling for the probability of pathogenicity at 0.50 for variants in the splice acceptor and splice donor of this exon. However, in contrast to *BRCA1* exons 9 and 10, protein‐truncating variants in *BRCA2* exon 12 are still expected to be pathogenic.

### Combining Prior Probabilities of Pathogenicity from Missense Substitution Severity and Spliceogenicity

Generating an overall prior probability in favor of pathogenicity for sequence variants falling in and around the protein coding exons of *BRCA1 and BRCA2* requires considering the priors from analyses of missense substitution severity, potential damage to wild‐type splice junctions, and potential creation of de novo splice donors. To do this, we look at a sequence variant from each of these perspectives and then assign the variant the highest prior probability in favor of pathogenicity generated by any one of the analyses. The resulting prior probabilities predicted on both missense and splicing analysis are available online for all possible single‐nucleotide substitutions in and around the protein coding exons of *BRCA1* and *BRCA2* at http://priors.hci.utah.edu/PRIORS.

### Variants Added to the Ex‐UV Database

In our previous effort to populate the *BRCA1/2* Ex‐UV LOVD with *BRCA1/2* missense substitutions that had previously published integrated evaluations [Vallée et al., 2012], we excluded certain missense substitutions from the database because they had (potentially) non‐negligible prior probabilities in favor of pathogenicity from a splice effects point of view. Here, we have reassessed those missense substitutions, plus a number of sequence variants falling on the intronic side of the splice junction consensus regions that have published observational data appropriate for use in an integrated evaluation. As a result, a total of 77 reanalyzable variants were added to the *BRCA1* and *BRCA2* Ex‐UV database.

Among these, we find 20 missense substitutions and 19 intronic splice region substitutions previously reported as neutral; 38 of these remain in either class 1 or class 2 (not pathogenic or likely not pathogenic). *BRCA1* c.5467+5G>C moved from class 2 (likely not pathogenic) [Whiley et al., 2011] to class 3 (uncertain) due to the sequence analysis based prior probability in favor of pathogenicity changing from the published value of 0.26–0.34 (see Table 6).

**Table 6:** *BRCA1* and *BRCA2* Sequence Variants Reclassified as a Consequence of Updated Prior Probabilities of Pathogenicity

Gene	HGVS (nucleotide)	HGVS (amino acid)	BIC	Motif location	*Z*‐score category	Published prior	Splicing prior	Missense prior	Selected prior	
*BRCA1*	c.4479_4484+2dup^a^	N/A	IVS14+2ins8	Donor	*Z* < −2.0	0.26	0.97	N/A	0.97	
	mRNA analysis: 8 bp retention of intron 14 [Whiley et al., 2011]									
*BRCA1*	c.4868C>G	p.A1623G	A1623G	De novo donor	−2 ≤ *z* < 0	0.01	0.64^b^	0.02	0.64	
	mRNA analysis: 119 bp deletion of exon 16, variant also present in wild‐type transcripts [Walker et al., 2010]									
*BRCA1*	c.5278‐14C>G	N/A	IVS20‐14C>G	Acceptor	*Z* > +0.5	0.26	0.04	N/A	0.04	
	mRNA analysis: no aberration detected [Spearman et al., 2008]									
*BRCA1*	c.5467+5G>C	N/A	IVS23+5G>C	Donor	−2 ≤ *z* < 0	0.26	0.34	N/A	0.34	
	Exon 23 deletion [Whiley et al., 2011]									
*BRCA2*	c.632‐16A>C	N/A	IVS7‐16A>C	Acceptor	Increased MES score	0.26	0.04	N/A	0.04	
	mRNA analysis: no aberration detected [Thomassen et al., 2012]									

Gene	HGVS (nucleotide)	Published source of observational data	Cosegregation Bayes score	Pathology LR^b^	Co‐occurrence LR	Family history LR	Published posterior	Recalculated posterior	IARC class^c^	Class description
*BRCA1*	c.4479_4484+2dup^a^	Whiley et al. (2011)	0.95	2.58	1	1	0.46	0.988	4	Likely pathogenic
*BRCA1*	c.4868C>G	Walker et al. (2010)	27.72	1.23	1.09	10.47	0.80	0.999	5	Pathogenic
*BRCA1*	c.5278‐14C>G	Whiley et al. (2011)	1	1	1.58	0.14	0.07	0.009	2	Likely not pathogenic
*BRCA1*	c.5467+5G>C	Whiley et al. (2011)	0.73	0.18	1	1	0.045	0.063	3	Uncertain
*BRCA2*	c.632‐16A>C	Thomassen et al. (2012)	1	1	1.20	0.22	0.086	0.011	2	Likely not pathogenic

a This variant was originally reported as c.4484+2ins8; insertion is due to duplication of GGAAAGGT.

De novo prior upgraded to the next higher qualitative category because the *z*‐score is higher than that of the corresponding wild‐type donor.

b Pathology likelihood ratios (LRs) based on breast tumor ER, grade, or TN status and using revised estimates from Spurdle et al. (in press). Cosegregation, co‐occurrence, and family history LRs are from the cited source of observational data.

c Class strata as described in Plon et al. (2008).

We also find nine missense substitutions and 19 intronic splice region substitutions previously reported as pathogenic; all of these remain in class 4 (likely pathogenic) or class 5 (pathogenic).

Of eight previously reported as uncertain, four remain in class 3. Two moved up to class 4 or class 5 (likely pathogenic or pathogenic), and two moved down to class 2 (likely not pathogenic); data on which the reclassification of these four substitutions rest are summarized in Table 6.

We also added two unusual variants to the Ex‐UV database.

The *BRCA1* BRCT domain missense substitution p.V1736A. This variant was observed in a woman who also carried the *BRCA1* frameshift variant c.2457delC and who was diagnosed at the age of 28 years with stage IV papillary serous ovarian carcinoma. Initially, co‐occurrence between the *BRCA1* frameshift and this missense substitution was taken as strong evidence against pathogenicity of the variant. Subsequent review of medical and photographic records revealed that the patient had abnormalities including microcephaly, macrognathia, and developmental delay; moreover, the patient had a severe response to carboplatin treatment that was more reminiscent of the response seen for biallelic BRCA1‐mutant mice than heterozygous human patients [Domchek et al., 2013]. Following qualitative MMR UV classification rules, potential biallelic mutation carriers with very early‐onset disease and developmentally abnormal clinical features should not be counted for purposes of calculating a co‐occurrence likelihood ratio [Thompson et al., 2014]. Taking into account that this is an Align‐GVGD C65 missense substitution (prior probability = 0.81) and the segregation likelihood ratio from 10 pedigrees is 234:1 [Domchek et al., 2013], we calculate a posterior probability of pathogenicity of 0.999 (class 5, clearly pathogenic).

The *BRCA2* translation initiator substitution c.3G>A. This variant was treated as a frameshift mutation because (1) it is expected to inactivate the wild‐type translation initiator, (2) the next available AUG in the mRNA is out of frame, and (3) this out‐of‐frame AUG has been shown in transfection experiments to outcompete the next available in‐frame AUG [Parsons et al., 2013]. For purposes of including the *BRCA2* c.3G>A in the Ex‐UV database, we use the prior probability of 0.96 suggested by Thomassen et al. (2012).

## Discussion

The work described here follows a pattern used in Tavtigian et al. [2008] in which we (1) used a sequence analysis algorithm to define what we hypothesized would be an ordered series of increasingly severe grades of sequence variants, (2) used one kind of data—counts of observed sequence variants and underlying dinucleotide substitution rate constants—available for the sequence variants in those grades to generate evidence that risk actually increased in the expected order, and (3) used a second quasi‐independent kind of data—the FamHx‐LRs—to replicate the evidence that risk actually increased in the expected order and to measure the risk for individual grades (or pooled grades) in such a way that the point estimates could become prior probabilities in favor of pathogenicity for clinical analysis of individual sequence variants reported in *BRCA1* and *BRCA2*.

An unusual element of this work, shared with our earlier calibration of missense substitution severity [Tavtigian et al., 2008], is that the prior probability of pathogenicity estimates emerge through direct steps from sequence analysis to estimates of pathogenicity in humans. Thus, questions of what fraction of the sequence variants analyzed actually impact mRNA splicing, or the extent to which the resulting splice isoforms encode dysfunctional proteins, were not used to estimate the prior probabilities. Nonetheless, subject to the limitations that (1) sequence variants assessed in an individual paper are often selected through some kind of algorithm and thus do not necessarily represent a random draw from available variants that meet a particular sequence analysis criterion, (2) assays of alternate or aberrant splicing are rarely quantitative and often overestimate the relative abundance of exon‐skipping isoforms, and (3) alternate or aberrant splice isoforms sometimes result in in‐frame deletions that encode a functional protein, we do observe reasonable agreement between the prior probabilities of pathogenicity for individual sequence analysis defined grades and the rates of severe splice aberrations likely to encode dysfunctional proteins in those same grades.

The resulting calibration of MES is almost entirely utilitarian. We have updated our *BRCA1/2* prior probability model for missense substitutions, previously based on position in the proteins and a measure of missense substitution severity, to include damage to wild‐type splice junctions and probability to create exonic de novo splice donors. Thus, the model is more complete and covers silent substitutions, missense substitutions, and sequence variants in the proximal splice junction consensus sequences. In principle, the model should apply to other susceptibility genes, and our java implementation of this MES‐based algorithm can be applied to VCF files and therefore whole‐exome sequences. As in our previous work, it was reassuring to see that when we saw significant evidence for increasing risk along an ordered series of sequence variant grades in the first data analysis, we saw the same ordering in the analysis of summary family history likelihood ratios. It was also reassuring that failure to see evidence for risk due to potential de novo exonic splice acceptors by the ERS analysis (Fig. 4) was replicated by failure to see evidence for increased risk due to potential exonic splice acceptors by the summary family history likelihood ratio analysis. Of course, why we were unable to usefully predict substitutions that would create de novo splice acceptors remains an interesting question.

A recent study, which examined 272 variants in and around the splice junctions of *BRCA1* and *BRCA2* variants with the aim of establishing guidelines for transcript analysis in a clinical setting, supports our findings on MES's reliability, as does a larger study of genome‐wide splice consensus region variants [Houdayer et al., 2012; Jian et al., 2014]. From their dataset of sequence variants’ effects on splicing, Houdayer et al. (2012) were able to perform receiver operating characteristic (ROC) analyses on several splice junction analysis programs; they found that MES was the single best performing program, with an area under the ROC curve of 0.956 (an AUC of 1 means 100% specificity, 100% sensitivity). Furthermore, Houdayer et al. (2012) affirmed that current programs are ineffective at predicting the effects of sequence variation in candidate exonic splice enhancers and branch points. However, the guidelines that they proposed only cover damage to splice sites (their analysis pipeline started with a variant in a consensus site). They did not attempt to analyze sequence variation within exons that could create de novo splice junctions, and they have not used their dataset to determine prior probabilities in favor of pathogenicity—key steps that we take here and then add to the overall integrated evaluation of unclassified *BRCA* gene sequence variants. Still, the de novo donor analysis presented here requires a cautionary note. Even from the B1&2 68K data set, the number variants with family history data that fell into the moderate or increased de novo donor categories was very small, resulting in wide 95% confidence intervals (Table 4). While the ordered result that we obtained in Table 4 and the corresponding categories of Table 5 is reassuring, additional data gathered over the coming years may well refine the current point estimates and prior probabilities.

While we were considering which published *BRCA* gene sequence variants to move into the Ex‐UV database, we ran up against the philosophical question of what combinations of sequence analysis‐based prior probability and observation data (expressed as a LR in favor of pathogenicity) actually constitute an integrated evaluation. The general principle is that at least two kinds of data have to be combined. But there are circumstances under which observational data are so uninformative that one could question whether their use actually constitutes a data integration. An extreme example would be a single observation of a *BRCA* gene missense substitution in an individual who did not carry any pathogenic variant in the same gene, with no other observational data provided. This datum would nominally convert to a coobservation LR of 1.03; if that LR were combined with the sequence analysis‐based prior, it would essentially constitute conversion of the prior probability to a posterior probability via only negligible observational data. Referring to the recent calibration of sequence analysis based prior probabilities for the mismatch repair genes missene substitutions, the minimum and maximum possible priors were truncated at 0.10 and 0.90, respectively [Thompson et al., 2013bb]. For these two priors, observational data LRs would have to be ≤0.47 or ≥2.2, respectively, in order for the variants to reach IARC class 2 or IARC class 4. Rounding these values off, we believe that ≤0.5 or ≥2 are reasonable inner boundaries for the magnitude of observational data LR required to perform a valid integrated evaluation. Applying this criterion, 77 reanalyzable variants had sufficient observational data and were added to the Ex‐UV database; however, we found 17 published “integrated evaluations” that did not meet this criterion and were consequently excluded [Walker et al., 2010; Whiley et al., 2011; Thomassen et al., 2012; Whiley et al., 2014b].

We close with a bioinformatics challenge. There may remain discrete but small groups of *BRCA1*/*2* sequence variants to which we wrongly assign low prior probabilities in favor of pathogenicity. Start from the list of all possible single‐nucleotide substitutions to the open‐reading frames of *BRCA1* and *BRCA2*. Exclude *BRCA1/2* nonsense substitutions and missense substitutions that are likely to be pathogenic because of missense dysfunction to *BRCA1*s RING or BRCT domains, or *BRCA2*s PALB2‐binding domain, DNA‐binding domains, or exon 27 RAD51‐binding site. Exclude substitutions that are likely to damage a *BRCA1/2* wild‐type splice junction. Exclude substitutions that are likely to create a de novo splice donor within an exon of *BRCA1* or *BRCA2*. Now, from the remaining list of exonic *BRCA1/2* gene single‐nucleotide substitutions, identify an algorithmically defined subset of substitutions that confers a statistically significantly increased risk of breast or ovarian cancer. We will be pleased to help calibrate prior probabilities in favor of pathogenicity for sequence variants grouped by that algorithm and then add the new priors into an ever‐evolving prior probability structure.

## Supporting information

Disclaimer: Supplementary materials have been peer‐reviewed but not copyedited.

Table S1

Table S2

Table S3
